# Mimvec: a deep learning approach for analyzing the human phenome

**DOI:** 10.1186/s12918-017-0451-z

**Published:** 2017-09-21

**Authors:** Mingxin Gan, Wenran Li, Wanwen Zeng, Xiaojian Wang, Rui Jiang

**Affiliations:** 1 Department of Management Science and Engineering, Dongling School of Economics and Management, University of Science and Technology Beijing, Beijing, 100083 China; 2 Ministry of Education Key Laboratory of Bioinformatics; Bioinformatics Division, Department of Automation and Tsinghua National Laboratory for Information Science and Technology, Tsinghua University, Beijing, 100084 China; 3 State Key Laboratory of Cardiovascular Disease, Fu Wai Hospital, National Center for Cardiovascular Diseases, Chinese Academy of Medical Sciences and Peking Union Medical College, Beijing, 100037 China; 4 Institute for Data Science, Tsinghua University, Beijing, 100084 China

## Abstract

**Background:**

The human phenome has been widely used with a variety of genomic data sources in the inference of disease genes. However, most existing methods thus far derive phenotype similarity based on the analysis of biomedical databases by using the traditional term frequency-inverse document frequency (TF-IDF) formulation. This framework, though intuitive, not only ignores semantic relationships between words but also tends to produce high-dimensional vectors, and hence lacks the ability to precisely capture intrinsic semantic characteristics of biomedical documents. To overcome these limitations, we propose a framework called *mimvec* to analyze the human phenome by making use of the state-of-the-art deep learning technique in natural language processing.

**Results:**

We converted 24,061 records in the Online Mendelian Inheritance in Man (OMIM) database to low-dimensional vectors using our method. We demonstrated that the vector presentation not only effectively enabled classification of phenotype records against gene ones, but also succeeded in discriminating diseases of different inheritance styles and different mechanisms. We further derived pairwise phenotype similarities between 7988 human inherited diseases using their vector presentations. With a joint analysis of this phenome with multiple genomic data, we showed that phenotype overlap indeed implied genotype overlap. We finally used the derived phenotype similarities with genomic data to prioritize candidate genes and demonstrated advantages of this method over existing ones.

**Conclusions:**

Our method is capable of not only capturing semantic relationships between words in biomedical records but also alleviating the dimensional disaster accompanying the traditional TF-IDF framework. With the approaching of precision medicine, there will be abundant electronic records of medicine and health awaiting for deep analysis, and we expect to see a wide spectrum of applications borrowing the idea of our method in the near future.

## Background

Deciphering genetic basis of human inherited diseases is a fundamental task in human and medical genetics [1]. Typically, this task is done by applying linkage analysis to a pedigree or association study to a cohort to roughly locate genomic regions that are statistically associated with a disease of interest, and then experimentally verify functions of genes located in these regions [2, 3]. In order to effectively determine target genes in functional experiments, computational methods are often used to prioritize candidate genes based on the “guilt-by-association” principle [4] by making use of multiple genomic data sources, including gene expression [5], protein sequences [6], protein-protein interaction [7], gene ontology [8, 9], and many others [10–14]. The basic assumption in a guilt-by-association analysis is that genes associated with a disease share common functions, and thus exhibit common characteristics across a variety of genomic data sources. As such, one can infer the functional similarity of a candidate gene to a set of seed genes that are already known as associated with the disease under investigation and then use the resulting score to rank candidate genes. However, the essential prerequisite of known seed genes greatly restricts the scope of application of these methods, making them more suitable to study diseases whose genetic basis is partly known a priori.

To overcome this limitation, there have been quite a few studies make use of the human disease phenome, particularly, relationships between all human disease phenotypes [15]. With a reasonable extension of the “guilt-by-association” principle to assume that genes causative for phenotypically related diseases often share common functions, computational methods can “borrow” known disease genes from highly correlated disease phenotypes, hence enabling the prioritization analysis for diseases whose genetic basis can be completely unknown [16]. With the incorporation of more sophisticated statistical models or machine learning methods, the utilization of the human phenome has evidenced a wide range of applications in deciphering genetic basis of human diseases [17–21].

Most existing methods for inferring the human phenome start from the Online Mendelian Inheritance in Man (OMIM) database [22]. In a typical pipeline, one first extracts biomedical concepts (terms) from a OMIM record based on a standardized vocabulary such as the Unified Medical Language System (UMLS) [23], Medical Subject Headings (MeSH) [24], and Human Phenotype Ontology (HPO) [25]. Then, the frequency of occurrence of a term in a record is counted, yielding a statistic called the term frequency (TF) that indicates how important the term is in the record. Meanwhile, the negative logarithm of the occurrence frequency of a term in all records is measured, resulting in another statistic called the inverse document frequency (IDF) that represents whether the term provides concrete meaning. The product of these two statistics is often referred to as TF-IDF, which has been frequently used as a weighting factor in information retrieval and text mining [26]. Finally, with the TF-IDF of every term collected, one represents a record as a vector of TF-IDFs. The cosine of the angle between two vectors can then be calculated to measure the similarity between two disease phenotypes.

In natural language processing, the above pipeline belongs to a category of methods called “bag-of-words” (BOW), which can be traced back to 1950’s and is now known to have several obvious disadvantages [27]. For example, words are treated independently in the calculation of TF-IDF values, and thus the semantic relationships between words are completely ignored. As an extreme example, any permutation of words in a record yields an identical TF-IDF vector as that obtained from the original record. This certainly goes against the objective of text mining. Moreover, the number of concepts is normally large, and thus the dimension of a TF-IDF vector is commonly high. In such a high dimensional space, the characterization of the similarity between two vectors is itself a difficult problem. In the case that the vectors are used in a machine learning task, the dimensional disaster seems inevitable.

Recent advances in the computer science community have evidenced several efforts to overcome the limitations of the “bag-of-words” methods. For example, Mikolov et al. introduced the skip-gram model [28], which represented words as vectors in a low-dimensional space and enabled precise prediction of the context surrounding a word. This method is ultra-efficient in that a single computer can easily train more than 100 billion words in a single day. Furthermore, sophisticated techniques such as sub-sampling of frequent words and the negative sampling have also been incorporated into the skip-gram model, speeding up this method by one order of magnitude with improved accuracy. Le and Mikolov further proposed a framework called paragraph vector that extends the vector representation from a single word to a sentence or even a document [29]. Besides the merit characteristic of converting unstructured text into a low dimension vector, these methods also have the ability to understand semantic relationships between words. For example, in the vector space, “Madrid” – “Spain” + “France” is closest to “Paris”, meaning that the vector presentation precisely grasps the intrinsic semantic relationships of a country and her capital.

With the understanding of shortcomings of analyzing the human phenome based on the traditional methodology of TF-IDF and merit characteristics of the recent advances in vector presentation of text, we propose in this paper a method named mimvec to analyze the human phenome. Our method converts 24,061 OMIM records to low-dimensional numeric vectors (e.g., 100 dimensions) by customizing the paragraph vector methodology. We show that the resulting vector representation of OMIM records not only effectively enables the classification of diseases against genes records in the OMIM database, but also successes in the discrimination between diseases of different inheritance styles. We further calculate phenotype similarities between 7988 disease phenotypes and use this resource with multiple genomic data sources to prioritize candidate genes, yielding a novel method for fining disease genes that exhibits superior performance over existing ones. To facilitate applications of our method, we provide free downloads of pre-calculated vector presentations of 24,061 OMIM records at http://bioinfo.au.tsinghua.edu.cn/jianglab/mimvec.

## Results

### Overview of the proposed approach

**Fig. 1:**
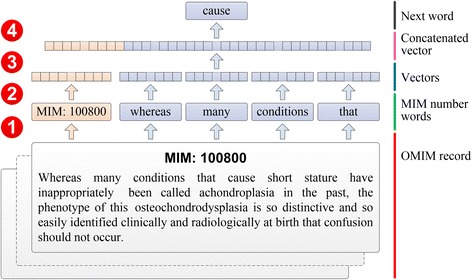
Overview of the proposed Mimvec method. Both the MIM number and the words in a sliding window are represented as low-dimensional numeric vectors, which are further concatenated to form features and used with a softmax function to predict the word followed. In the training phase, vectors are initialized at random, and OMIM records are fed sequentially to the model. The average log probability of all predictions and maximized via a stochastic gradient descent algorithm with backpropagation. When a model was well trained, vectors corresponding to MIM numbers are extracted

The proposed method, *mimvec*, customized a deep learning method in natural language processing called Paragraph Vector [29] to analyse OMIM records and converted them to low-dimensional numeric vectors. As illustrated in Fig. 1, we first identified the MIM number of an OMIM record from the NO field, and we extracted from the TX field a sequence of words, in the order as they appeared in the record. In this procedure, we discarded all section captions, punctuations and numbers. Then, we represented both the MIM number and the words as low-dimensional (e.g., one hundred) numeric vectors. Next, we concatenated vectors corresponding to the MIM number and a small number of words in a sliding window (e.g., of size five) to form a new vector. Finally, we used this vector to predict the word appearing immediately after the window. In this model, an OMIM record was identified by its own MIM number, and thus was represented by a distinct vector. A word, on the contrary, was often shared by a number of OMIM records, and thus the corresponding vector was also shared across records. In this sense, the vector corresponding to an OMIM record provided information specific to the record and enabled more precise analysis of the relationship between the words within the record. The prediction task was modeled as a multiclass classification problem and solved by adopting a neural network, which took the concatenated vector as input and produced the probability of a word via a softmax function. In order to train such a model, we first initialized all vectors at random and then fed OMIM records to the model and maximized the average log probability of all predictions via a stochastic gradient descent algorithm with backpropagation (see Methods for details). When a model was well trained, we extracted the vector corresponding to a MIM number to obtain the vector presentation of the corresponding OMIM record.

### Mimvec distinguishes phenotypes from genes

**Fig. 2:**
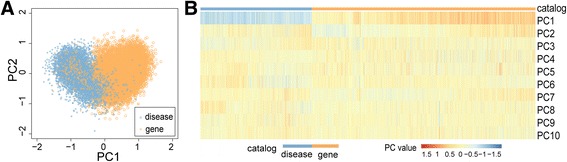
Mimvec distinguished phenotypes from genes. **a** Projection of OMIM records to a two-dimensional Euclidean space using the first two principle components shows that diseases can be well distinguished from genes. **b** The first component alone can already discriminate diseases from genes with high accuracy

We asked the question of whether the vector presentation of an OMIM record could capture its intrinsic semantic characteristics. To answer this question, we converted all the 24,061 OMIM records to 100-dimensional vectors, and we explored the possibility of distinguishing the 7988 phenotype records from the rest 16,073 gene records. We first applied a principle component analysis (PCA) to the matrix (24,061×100) containing vectors of all the records and visualized the results in a two-dimensional Euclidean space composed of the first two principle components (PC). As demonstrated in Fig. 2a, we find that dots corresponding to diseases can be well distinguished from those corresponding to genes, except for a small number of outliers. Moreover, if we project the dots to the first principle component (*x*-axis), diseases obviously have smaller coordinates than genes. To show this observation in a clearer way, we plotted a heatmap by using the first 10 principle components (Fig. 2b). It is clear that the first principle component alone can well distinguish diseases from genes (accuracy = 91.45%), suggesting that most information contributing to the characteristics of these two different categories of records is already captured by this component.

We then performed a binary classification of disease records against gene ones by using all elements in the 100-dimensional vectors as features. We evaluated three classifiers, logistic regression (LR), random forest (RF) and support vector machine (SVM), using 10-fold cross-validation and show the results in Table 1. From the table, we clearly see that all the three classifiers can well discriminate between these two catalogs. For example, the area under the receiver operating characteristic curve (AUC) for logistic regression is as high as 99.41%, and the classification accuracy (ACC) achieves 96.17%. Considering the situation of imbalance between the number of diseases (7988) and that of genes (16,073), we further calculated a criterion called balanced error rate (BER, the average of error rates for the two classes) and obtain a value of only 2.6%, again revealing the effectiveness of classifying diseases against genes using elements in the vector presentation as features.

**Table 1:** Classification of phenotypes against genes

	AUC (%)			ACC (%)			BER (%)		
	LR	RF	SVM	LR	RF	SVM	LR	RF	SVM
Mimvec (50)	99.31	99.20	99.34	97.52	96.49	97.80	2.97	4.29	2.74
Mimvec (100)	**99.41**	**98.92**	**99.31**	**97.83**	**96.17**	**97.78**	**2.60**	**5.19**	**2.75**
Mimvec (150)	99.35	98.79	99.34	97.73	95.21	97.69	2.71	6.41	2.83
Mimvec (200)	99.43	98.67	99.26	97.75	94.43	97.67	2.65	7.99	2.85
Mimvec (250)	99.42	98.71	99.33	97.73	94.04	97.86	2.75	8.55	2.64
Mimvec (300)	99.36	98.50	99.17	97.70	93.59	97.60	2.75	9.32	2.91

Disease and gene records can be well distinguished by vector representations of the records

Bold numbers highlight performance achieved at the default setting (100 dimensions)

We further tried different numbers of dimensions in the vector presentation and found the difference in the results is negligible for logistic regression and support vector machine. The performance of random forest, however, tends to drop with the increase of the number of dimension, especially for BER, suggesting that low dimensional vectors are preferred.

All these results support the conclusion that the vector presentation of OMIM records can indeed capture intrinsic characteristics of the records. Furthermore, the number of dimensions of the presentation is not critical for the capture of information implicated in the records.

### Mimvec distinguishes diseases of different inheritance styles

We further asked the question of whether the vector presentation of phenotypes could distinguish diseases of different inheritance styles. To answer this question, we identified 1853 autosomal dominant diseases (MIM: 1xxxxx) and 1547 autosomal recessive diseases (MIM: 2xxxxx) from the OMIM database, and again applied the three classifiers (LR, RF and SVM) to classify autosomal dominant diseases against autosomal recessive ones. Results of the leave-one-out cross-validation experiments, as shown in Table 2, give us a positive answer to this question. For example, when presenting a phenotype as a 100-dimensional vector, the AUC, ACC and BER for logistic regression are 89.85, 83.21 and 17.05%, respectively. The other two methods also achieve reasonably high performance (RF: AUC = 87.29%, ACC = 79.32% and BER = 21.59%; SVM: AUC = 89.80%, ACC = 83.18% and BER = 17.10%). These results reveal the effectiveness of the vector presentation in the classification of diseases of different inheritance styles. Furthermore, we tried different numbers of dimensions in the vector presentation and found the performance of LR and SVM tends to improve with the increase of the number of dimensions, while that of RF tends to drop, though the change is itself small (Table 2).

**Table 2:** Classification of autosomal dominant diseases versus autosomal recessive ones

	AUC (%)			ACC (%)			BER (%)		
	LR	RF	SVM	LR	RF	SVM	LR	RF	SVM
Mimvec (50)	87.75	86.04	87.56	80.97	78.74	80.91	19.37	22.03	19.46
Mimvec (100)	**89.85**	**87.29**	**89.80**	**83.21**	**79.32**	**83.18**	**17.05**	**21.59**	**17.10**
Mimvec (150)	90.57	86.93	89.95	83.47	78.76	82.76	16.72	22.19	17.37
Mimvec (200)	90.82	85.95	91.12	85.00	77.68	84.91	15.14	23.33	15.33
Mimvec (250)	90.88	86.00	91.71	84.47	78.56	85.50	15.69	22.57	14.68
Mimvec (300)	91.02	86.00	91.62	84.74	77.68	85.06	15.46	23.58	15.15

Diseases of different inheritance styles can be well distinguished by vector representations of the records

Bold numbers highlight performance achieved at the default setting (100 dimensions)

We then identified 48 immune diseases and 263 neurological disorders according to the Genetic Association database [30], and we also applied the three classifiers (LR, RF and SVM) to discriminate these two catalogs of diseases. Results of the leave-one-out cross-validation experiments, as shown in Table 3, demonstrate the possibility of solving this binary classification problem using the vector presentation of disease phenotypes as features. For example, with 100-dimensional vectors, the AUC, ACC and BER for SVM are 85.62, 86.13 and 29.14%, respectively. The other two methods also achieve reasonably high AUC and ACC (LR: AUC = 80.63%, ACC = 81.94%; RF: AUC = 78.20%, ACC = 85.16%). However, we notice that BER for RF is only 44.57%, suggesting that this classifier tends to assign wrong label to one of the catalogs (i.e., immune diseases). We guess the phenomenon is due to the imbalance of the training samples (48 versus 263). We further tried vectors of different dimensions and found the performance of LR and RF tends to drop with the increase of the number of dimensions. For SVM, the classification performance is quite stable for different number of dimensions (Table 3).

**Table 3:** Classification of Immune diseases versus neurological disorders

	AUC (%)			ACC (%)			BER (%)		
	LR	RF	SVM	LR	RF	SVM	LR	RF	SVM
Mimvec (50)	77.94	83.49	82.99	86.77	86.45	88.06	25.27	38.56	28.88
Mimvec (100)	**80.63**	**78.2**	**85.62**	**81.94**	**85.16**	**86.13**	**32.49**	**44.57**	**29.14**
Mimvec (150)	71.98	72.81	80.67	74.52	84.19	85.16	38.61	46.89	30.59
Mimvec (200)	76.11	78.89	85.83	74.84	84.19	89.03	33.18	48.63	24.81
Mimvec (250)	65.73	76.63	84.6	68.39	84.52	87.42	41.35	47.57	28.38
Mimvec (300)	61.14	72.83	84.08	61.14	72.83	84.08	46.94	46.51	28.08

Diseases of different mechanisms can be well distinguished by the vector representation of the records

Bold numbers highlight performance achieved at the default setting (100 dimensions)

### Mimvec links phenotypes to causative genes

A fundamental problem in human genetics is to link phenotypes to genotypes. In medical genetics, it is of great importance to identify genes responsible for a disease phenotype. This is typically done by applying linkage analysis or association studies to identify genomic regions that show strong association with a specific disease of interest and then prioritizing candidate genes located in these regions by making use of such genomic data sources as gene expression [5] and protein-protein interaction [7]. With disease phenotypes and genes represented as vectors by our unsupervised deep learning approach, we explore the possibility of linking a disease phenotype to its causative genes by using their vectors alone.

**Fig. 3:**
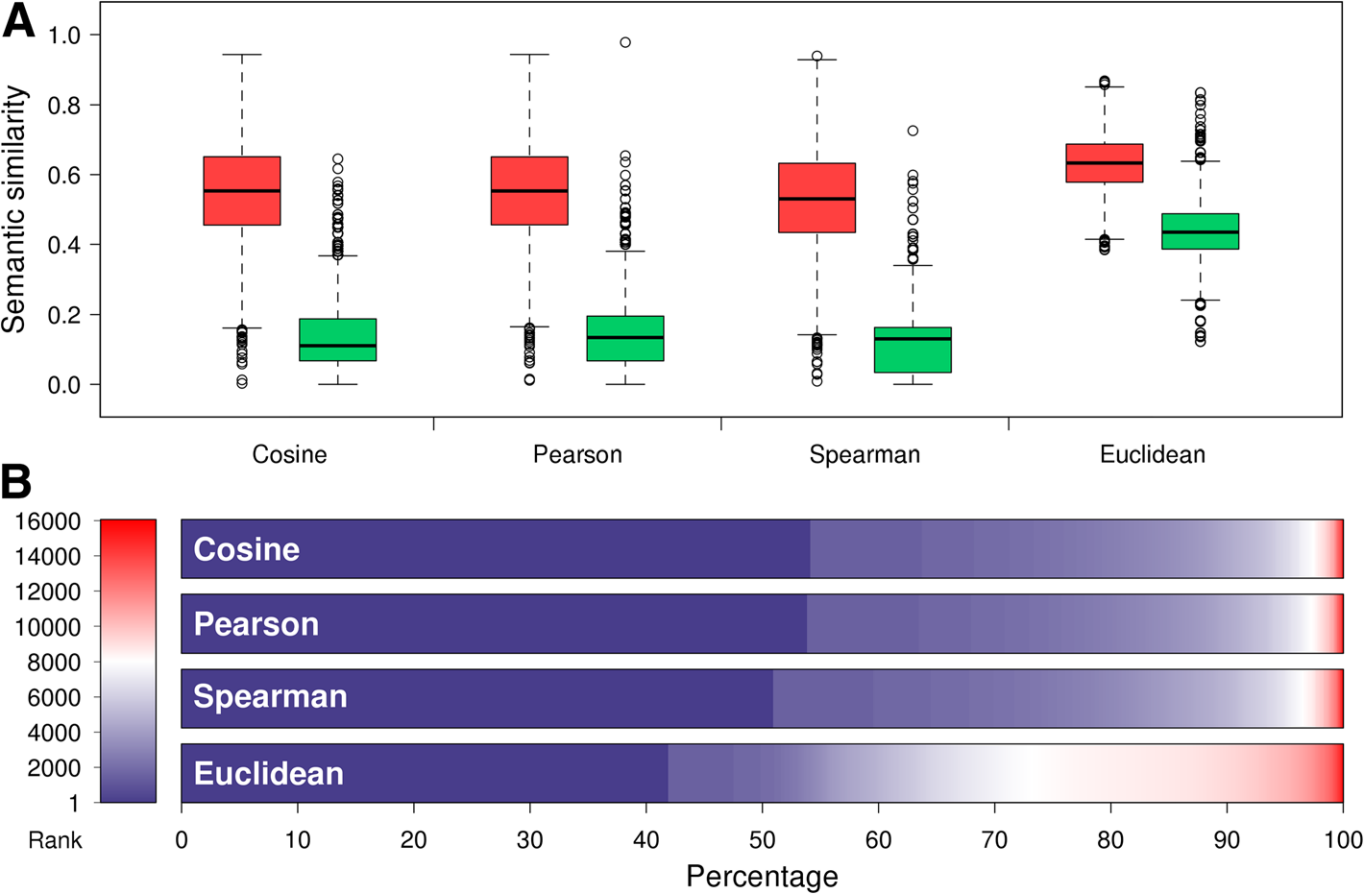
Mimvec links phenotypes to causative genes by semantic similarity. **a** Distribution of similarities between diseases and their known causative genes exhibit an obvious positive shift against that between diseases and genes selected at random. **b** Most known disease genes are ranked among top positions

From the OMIM database, we identified 4397 associations between 3798 diseases and 2944 genes. We then quantified the similarity between every pair of disease and gene by using the cosine measure, and we plotted distributions of the resulting similarities. As shown in Fig. 3a, the distribution of similarities between diseases and their known causative genes exhibit an obvious positive shift against that between diseases and genes selected at random, suggesting the possibility of utilizing this cosine measure as a score to distinguish true causative genes from irrelevant ones. With this understanding, we conducted a leave-one-out experiment to simulate the ambitious goal of identifying causative genes for a specific disease. In detail, for each of the 4397 known associations between a disease and a gene, we ranked the gene against other genes according to the cosine score (the larger the better), with genes known as associated with the disease excluded. As shown in Fig. 3b, 2379 (54.11%) known disease genes are ranked first, 422 (9.60%) ranked second, 197 (4.48%) ranked third. In contrast, with a random guess procedure, one could only expect to see 0.2736 (4397/16,073) known disease genes ranked first. In other words, prioritizing candidate genes according to the cosine similarity score yields a fold enrichment of more than 8695 (2379/0.2736), strongly suggesting the effectiveness of this method for finding disease genes.

We further repeated the above experiments with the use of three alternative similarity measures (Pearson’s correlation coefficient, Spearman’s correlation coefficient, and normalized Euclidean similarity). As shown in Fig. 3a, for all the three measures, the distribution of similarities between diseases and their known causative genes also exhibits an obvious positive shift against that between diseases and genes selected at random. As shown in Fig. 3b, all the three similarity scores are also capable of distinguishing causative genes from irrelevant ones. Moreover, we notice that the cosine and Pearson’s correlation coefficient measures yield similar performance, which is obviously higher than that of Spearman’s correlation coefficient and normalized Euclidean similarity.

### Mimvec bridges phenotype similarity and gene functional overlap

Most computational methods for finding disease genes obey the guilt-by-association principle, which assumes that genes associated with a disease would have similar functions [4]. A more general assumption is that genes associated with phenotypically similar diseases exhibited functional similarities across different genomic data sources [19]. This assumption has become the basis for designing gene prioritization methods, with such successful stories as PRINCE [21], pgFusion [18], pgWalk [17] and many others [15]. With our method to characterize phenotype similarity based on vector representations of phenotypes with the use of the cosine measure or the alike, we explore whether the resulting phenotype similarity between diseases also implies genotype overlap.

**Fig. 4:**
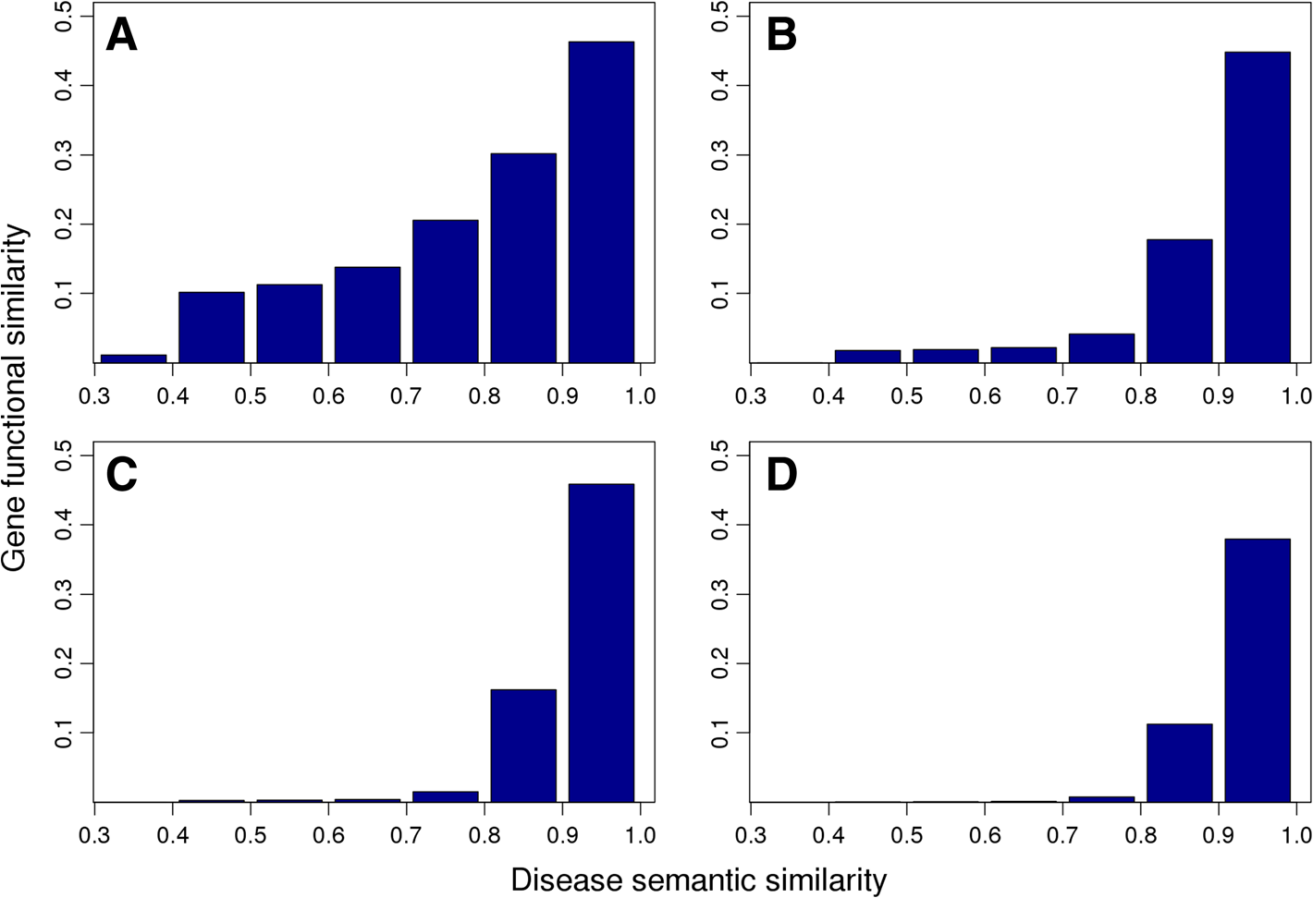
Mimvec bridges disease semantic similarity and gene functional similarity. There exist strong correlations between the phenotype similarity and the genotype overlap. Four genomic data sources are used, include **a** gene expression, **b** protein string, **c** protein-protein interaction. **d** gene ontology (biological process)

We identified 4593 associations between 3921 diseases and 3023 genes using the tool BioMart [31]. For every pair of diseases, we measured their phenotype similarity based on the vector presentation (100 dimension) using the cosine score, and we measured their genotype overlap as the average pairwise similarity scores of their associated genes under a genomic data source. Particularly, we adopt four types of genomic data (RNA-seq, protein sequence, protein-protein interaction, and gene ontology, as described in Methods). We then partitioned the phenotype similarity scores into 10 bins of equal size, identified disease pairs belonging to each bin, and calculated the average genotype similarity of disease pairs in each bin, as shown in Fig. 4. From this figure, we observe strong correlations between the phenotype similarity and the genotype overlap. Taking gene expression derived from RNA-seq data as an example, it is shown that the genotype similarity increases with the increase of the phenotype similarity (Fig. 4a). Particularly, for disease pairs with very weak phenotype similarity (< 0.3), their genotype similarity is also very weak (near zero, not shown). For disease pairs with strong phenotype similarity (0.9 ~ 1.0), their genotype similarity is also strong (0.4630 on average). In the middle of the spectrum, for disease pairs with medium phenotype similarity (0.5 ~ 0.6), their genotype similarity is also at the medium level (0.1378 on average). We further regressed the mean genotype similarity of each bin against the corresponding mean phenotype similarity. For the other four genomic data sources, we observe similar patterns (Fig. 4 b-d). These results clearly suggest that genes associated with phenotypically similar diseases indeed exhibit functional similarities across different gnomic data sources. We further regressed the mean genotype similarity of each bin against the corresponding mean phenotype similarity. Results show that the coefficients of determination (*r* ^2^) are 0.9621 for the gene expression, 0.7573 for protein sequence, 0.7140 for protein-protein interaction, and 0.6860 for gene ontology. These results further suggest that the phenotype similarity derived from the vector presentation implies the genotype overlap.

### Mimvec enables the prioritization of candidate genes

With the assumption that genes associated with phenotypically similar diseases exhibited functional similarities across different genomic data sources being validated, we further implemented a random walk with restart model (see Methods for details) to prioritize candidate genes to demonstrate how the vector presentation of phenotypes can be used to facilitate the identification of disease genes.

We performed three large-scale leave-one-out cross-validation experiments to validate the effectiveness of this method using the 4593 associations between 3921 diseases and 3023 genes. We fist simulated the situation of a traditional linkage analysis or association study, in which the objective was to prioritize candidate genes in a linkage interval. In each validation run, we focused on one known disease-gene pair in an annotated association and looked at whether our method could correctly identify the gene from a set of control genes that were located within a 10 Mb region centred at the test gene (i.e., the gene associated with the disease), and ranked the test gene against the control genes using our method. In this procedure, we removed all annotated associations regarding the query disease to simulate the circumstance that the genetic basis of the query disease was completely unknown.

We derived three criteria to quantify the performance of our method. Dividing the number of test genes ranked first by the total number of candidate genes, we obtained a criterion called the top ranked test genes (TOP). Dividing the rank of a test gene by the total number of test and control genes in a validation run, we obtained the rank ratio of the test gene. Averaging rank ratios of all test genes, we obtained a criterion called the Mean Rank Ratio (MRR). At a certain threshold of the rank ratio, we defined the sensitivity and the specificity as the fraction of test and control genes ranked above and below the threshold, respectively. Varying the threshold, we plotted the rank operating characteristic (ROC) curve (sensitivity versus 1-specificity) and further calculated the area under this curve as a criterion called the AUC score.

**Fig. 5:**
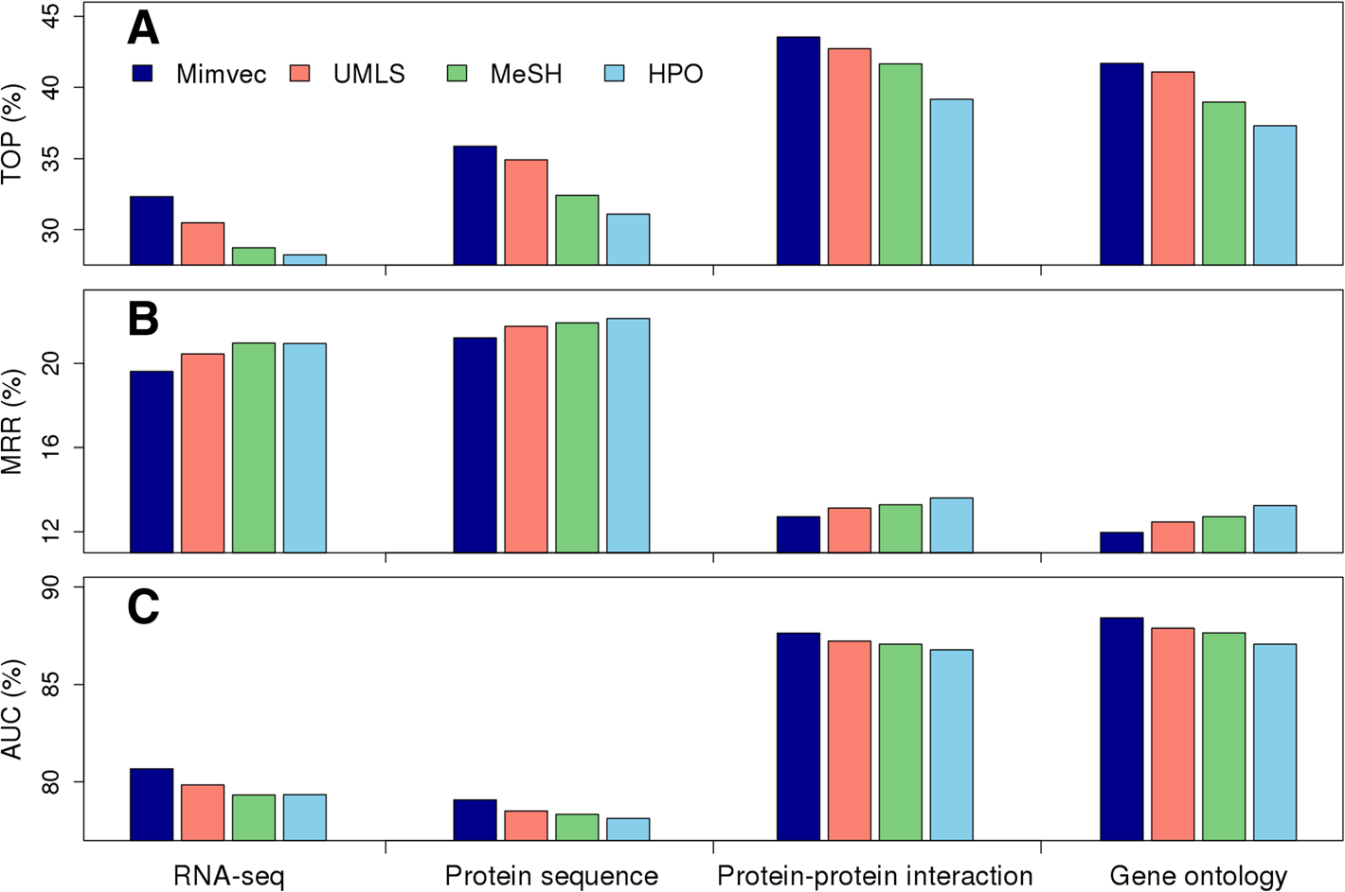
Mimvec enables the prioritization of candidate disease genes in linkage interval cross-validation experiments. All the three evaluation criteria (**a**. TOP, **b**. MRR, and **c**. AUC) support that the performance of the four phenotype similarity measure can be sorted as mimvec > UMLS > MeSH > HPO

As shown in Table 4 and Fig. 5, TOP, MRR and AUC for validation experiment against a linkage interval with the use of phenotype similarity derived from the vector presentation (mimvec, 100-dimension) and gene similarity derived from RNA-seq data are 32.33, 19.62 and 80.67% respectively. In contrast, when using phenotype similarity derived from ULMS by analysing TF-IDF of biomedical concepts (Methods), the TOP, MRR and AUC are only 30.48, 20.45 and 79.84%, respectively. When using the phenotype similarity derived from MeSH, the TOP, MRR and AUC are only 28.72, 20.97 and 78.35%, respectively. When using the phenotype similarity derived from HPO, the TOP, MRR and AUC are only 28.22, 20.95 and 79.34%, respectively. Since the genotype similarity data are the same in the above comparison, these comparisons suggest that the vector presentation of phenotypes, though does not resort to any prior knowledge about biology and medicine, is superior to the TF-IDF counterparts that utilize UMLS, MeSH and HPO. We further repeated the above experiments by using gene similarity derived from protein sequence, protein-protein interaction data and gene ontology, and the results give us similar conclusion. Particularly, when fixing a phenotype similarity measure, performance of the four genomic data can be sorted as protein-protein interaction > gene ontology > protein sequence > gene expression. When fixing a genotype similarity measure, performance of the four phenotype similarity measure can be sorted as mimvec > UMLS > MeSH > HPO.

**Table 4:** Performance of the random walk model with different measures of phenotype similarity and gene similarity in the leave-one-out cross-validation experiments

		TOP (%)				MRR (%)				AUC (%)			
		RSEQ	PSEQ	STRG	GOBP	RSEQ	PSEQ	STRG	GOBP	RSEQ	PSEQ	STRG	GOBP
Linkage Interval	Mimvec	32.33	35.88	43.54	41.69	19.62	21.22	12.72	11.97	80.67	79.07	87.65	88.41
	UMLS	30.48	34.92	42.72	41.08	20.45	21.77	13.13	12.47	79.84	78.52	87.24	87.90
	MeSH	28.72	32.42	41.65	38.99	20.97	21.93	13.29	12.72	79.32	78.35	87.08	87.66
	HPO	28.22	31.09	39.19	37.32	20.95	22.14	13.61	13.25	79.34	78.14	86.79	87.08
Nearest Neighbor	Mimvec	40.37	44.11	54.19	51.19	19.19	20.89	12.38	11.59	81.13	79.46	88.02	88.81
	UMLS	37.93	43.04	52.99	50.03	20.07	21.50	12.80	12.10	80.24	78.85	87.60	88.29
	MeSH	35.53	40.00	51.40	47.72	20.54	21.60	12.90	12.35	79.76	78.75	87.43	88.04
	HPO	34.44	37.77	48.29	44.83	20.53	21.77	13.27	12.90	79.77	78.57	87.12	87.29
Random Control	Mimvec	44.33	50.36	59.87	57.04	17.72	20.34	11.66	10.83	82.60	80.01	88.74	89.57
	UMLS	41.48	46.94	57.65	54.34	18.98	20.91	12.17	11.37	81.33	79.43	88.61	89.02
	MeSH	38.71	44.74	55.32	51.86	19.59	21.05	12.32	11.63	80.72	79.30	87.43	88.76
	HPO	37.21	41.96	52.23	44.83	19.56	21.26	12.65	12.14	80.75	79.08	87.75	88.24

*Mimvec* phenotype similarity derived from the vector presentation of phenotypes (100 dimension), *UMLS* phenotype similarity derived from the Unified Medical Language System, *MeSH* phenotype similarity derived from the Medical Subject Headings, *HPO* phenotype similarity derived from the Human Phenotype Ontology, *RSEQ* gene similarity derived from the RNA-seq data, *PSEQ* gene similarity derived from the protein sequence data, *STRG* gene similarity derived from the protein-protein interaction data, *GOBP* gene similarity derived from the gene ontology (biological process)

The number of control genes in a linkage interval may have variation, thereby introducing biases in assessing the capability of a method in enriching test genes at top positions (e.g., ranking a test gene among top 10 against 20 control genes is much easier than ranking it among top 10 against 100 control genes). We therefore performed another validation experiment (i.e., nearest neighbor) by ranking each test gene against 99 control genes that were closest to the test gene in the same chromosome. From the results shown in Table 4, we draw the same conclusion as we have for linkage interval. Briefly, when fixing a genotype similarity measure, performance of the four phenotype similarity measure can be sorted as mimvec > UMLS > MeSH > HPO, no matter which evaluation criterion is used.

We further simulated the situation of exome sequencing studies, in which genetic variants are sequenced across the whole exome. In each validation run, we focused on one disease-gene pair in an annotated association and ranked the test gene against a set of 99 control genes that were selected at random from the entire genome. From the results shown in Table 4, we draw the same conclusion as we have for linkage interval and nearest neighbor. That is, when fixing a genotype similarity measure, the performance of the four phenotype similarity measure can be sorted as mimvec > UMLS > MeSH > HPO.

The above results are obtained by representing a phenotype as a vector of 100 dimensions. To study the possible influence of the number of dimensions to the performance of our prioritization method, we varied the vector size from 50 to 300 with step 50 and repeated the cross-validation experiments. As shown in Table 5, in general, the performance is quite robust for different number of dimensions, since the evaluation criteria do not vary much with the variation of the numbers of dimensions. In more detail, a relatively small number of dimensions (e.g., 50) can already give us reasonably good performance (higher than UMLS, MeSH and HPO). A relatively large number of dimensions does not show much help in improving the performance. When the number of dimensions is greater than 200, we even observe a drop of the performance. Considering that the number of parameters in the neural network increases linearly with the number of dimensions, and thus the computational burden also increases, a medium number of dimensions (e.g., 100) seems already a good choice.

**Table 5:** Robustness of the phenotype similarity derived from phenotype vectors of different dimensions

		TOP (%)				MRR (%)				AUC (%)			
		RSEQ	PSEQ	STRG	GOBP	RSEQ	PSEQ	STRG	GOBP	RSEQ	PSEQ	STRG	GOBP
Linkage Interval	Mimvec (50)	31.09	34.57	42.37	40.47	19.64	21.37	12.89	11.84	80.66	78.91	87.47	88.54
	Mimvec (100)	32.33	35.88	43.54	41.69	19.62	21.22	12.72	11.97	80.67	79.07	87.65	88.41
	Mimvec (150)	32.09	35.52	43.22	41.08	19.95	21.46	12.91	12.31	80.34	78.82	87.45	88.06
	Mimvec (200)	32.33	35.97	43.15	41.56	20.10	21.59	13.06	12.43	80.19	78.69	78.30	87.94
	Mimvec (250)	31.53	35.29	42.93	41.11	20.29	21.62	12.98	12.45	80.00	78.67	87.39	87.92
	Mimvec (300)	32.05	35.38	43.20	41.13	20.22	21.83	13.05	12.61	80.07	78.45	87.31	87.76
Nearest Neighbor	Mimvec (50)	38.36	42.39	52.21	49.88	19.19	21.05	12.56	11.43	81.13	79.30	87.84	88.96
	Mimvec (100)	40.37	44.11	54.19	51.19	19.19	20.89	12.38	11.59	81.13	79.46	88.02	88.81
	Mimvec (150)	40.50	44.22	54.15	51.32	19.50	21.13	12.53	11.88	80.81	79.22	87.86	88.51
	Mimvec (200)	40.74	44.35	53.69	51.36	19.68	21.25	12.69	12.06	80.63	79.10	87.70	88.33
	Mimvec (250)	39.47	43.81	53.52	50.95	19.84	21.27	12.61	12.04	80.47	79.08	87.79	88.84
	Mimvec (300)	40.21	43.97	53.78	50.84	19.78	21.47	12.66	12.21	80.52	79.28	87.74	88.18
Random Control	Mimvec (50)	42.72	48.01	58.61	54.78	17.83	20.43	11.87	10.68	82.50	79.91	88.53	89.73
	Mimvec (100)	44.33	50.36	59.87	57.04	17.72	20.34	11.66	10.83	82.60	80.01	88.74	89.57
	Mimvec (150)	44.46	49.50	59.81	56.50	18.08	20.59	11.86	11.08	82.24	79.76	88.54	89.32
	Mimvec (200)	44.68	49.40	59.50	55.93	18.34	20.71	11.98	11.26	81.99	79.64	88.43	89.64
	Mimvec (250)	43.94	49.25	59.18	55.91	18.48	20.71	11.95	11.22	81.84	79.64	88.45	89.68
	Mimvec (300)	44.11	48.88	59.02	55.76	18.37	20.95	11.97	11.43	81.95	79.39	88.44	88.96

Mimvec (50 ~ 300): phenotype similarity derived from phenotype vectors of different dimensions

## Discussion

We conjecture that the success of our method can be attributed to the combination of the following aspects. First, our model considers local semantic relationships between words instead of treating words as independent units. From the viewpoint of natural language processing, relying on a standardized vocabulary (e.g., UMLS, MeSH and HPO) and the TF-IDF measure to analyze OMIM records belongs to a class of methods called “bag-of-words” (BOW), which ignores relationships between words and thus cannot capture semantic meaning of nearby words. In contrast, our method overcomes this limitation by predicting a word using its predecessors, and thus implicitly takes semantic relationships between words into consideration. Second, our model represents a record as a low-dimensional dense vector, while traditional methods based on TF-IDF describes a record as a high-dimensional sparse vector. However, a large number of dimensions usually leads to a disaster in machine learning, yielding such hard tasks as feature selection. Besides, the precise measure of the similarity between two vectors in a high-dimensional space is itself a difficult problem, no mention the fact that the space is very parse.

The main weakness of our vector representation method is that a dimension does not have the concreate meaning. In methods based on TF-IDF, a dimension corresponds to a term or concept in a standardized vocabulary, and thus it is convenient to explain the meaning of an element in a TF-IDF vector. Our method, however, embeds and compresses a record into a low dimensional vector, and hence the meaning of a dimension is not clear. With understanding, it might be worth pursuing to seek for a methodology similar to deconvolution to dissect the meaning of a vector. Another possible improvement of our method is to stand on the shoulders of already established fruitful biomedical knowledge. Although we have demonstrated the effectiveness of the vector representation without the use of any prior information about biomedical concepts, starting from scratch certainly wastes such knowledge that has been accumulated for a long period of time. In this sense, it might be worth pursuing to incorporate biomedical knowledge into our approach to further improve its effectiveness. Finally, the methodology of representing a document as a vector is not specific to the analysis of OMIM records. With the coming of precision medicine, there are abundant electronic records of medicine and health awaiting for deep analysis. We expect to see a wide range of applications borrowing the idea of our method in the near future.

## Conclusions

In this paper, we have proposed a deep learning approach named mimvec to analyse the OMIM database, with particular emphasis on the human phenome. We have shown that the unsupervised conversion of OMIM records to low-dimensional vectors effectively enables the classification of diseases against genes, the discrimination between diseases of different inheritance styles, and the prioritization of candidate genes. When utilized with multiple genomic data sources, the similarity measure derived from vector presentation of phenotypes with no prior knowledge exhibits superior performance over traditional measures derived from ULMS, MeSH and HPO in a model for prioritizing candidate genes.

## Methods

### Data sources

We extracted 24,061 records from the OMIM database (accessed in April 2015) and identified 7988 disease phenotypes and 16,073 genes, represented by MIM numbers. We identified 20,327 genes from the Ensembl database (accessed in November 2015), represented by Ensembl gene ID. We extracted 4606 associations between 3933 diseases and 3028 genes by using the tool BioMart [31]. On average each disease was associated with 1.17 genes, and each gene was relevant to 1.52 diseases. We downloaded raw sequencing data of 503 RNA-seq experiments from the ENCODE projects and calculated expression levels (FPKM) of the 20,327 genes by using the standard Tophat and Cufflinks pipeline. We extracted sequences of 20,272 human proteins from the Swiss-Prot database (release 2014_01). We extracted 403,514 interactions between 13,747 human proteins from the STRING database (version 9.1). We extracted the biological process domain of the gene ontology and downloaded associated annotations for 15,602 human genes (both released on 2014–11-22).

### Vector presentation of phenotypes based on a standardized vocabulary

We adopted a text mining technique to characterize an OMIM record of human disease phenotype by using a standardized vocabulary. First, we splinted sentences in the TX and CS fields of a record into words, and we mapped these words onto UMLS concepts by using the program MetaMap [32] (Version 2016 V2), obtaining 8446 concepts for describing human disease phenotypes. For each of these concepts, we counted its occurrence frequency in the record to obtain a statistic called term frequency (TF), calculated the negative logarithm of its occurrence frequency in all OMIM records to obtain a statistic called document frequency (IDF), and derived a quantity called TF-IDF as the product of the TF and IDF values. Concatenating this quantity for all concepts together, we obtained a TF-IDF vector to characterize the record based on UMLS. Second, with a similar procedure, we obtained 4097 concepts by using MeSH as the standard vocabulary and characterized an OMIM record using a TF-IDF vector of these terms. Third, focusing on HPO and associated annotations for 6708 human disease phenotypes [25], we collected 11,813 concepts in the ontology and characterized a phenotype using a numeric vector of such number of dimensions. An element in such a vector was the information content of the corresponding concept, calculated as the negative logarithm of its occurrence frequency in the annotations. Considering the directed acyclic graph (DAG) structure of HPO, we added the occurrence frequency of a concept to its parents recursively.

### Vector presentation of phenotypes using paragraph vector

Let **M** = (*m*_*ij*_)_*m* × *d*_ be the vector presentation of all OMIM records, where *m* is the number of records and *d* the number of dimension of a vector. Each row of this matrix, **m**_*i*_ = (*m*_*ij*_)_1 × *d*_, corresponds to the vector presentation of a record *i*. Let **W** = (*w*_*ij*_)_*w* × *d*_ be the vector presentation of all words in OMIM records, where *w* is the number of words and *d* the number of dimension of a vector. Each row of this matrix, **w**_*i*_ = (*w*_*ij*_)_1 × *d*_, corresponds to the vector presentation of a word *i*. Given a sequence of words in a window of size *k* starting from the *t*-th word in a record *r*, represented by corresponding vectors ${\mathbf{w}}_t^r,\dots, {\mathbf{w}}_{t+k-1}^r$, we denote the log likelihood of predicting the word ${\mathbf{w}}_{t+k}^r$ as

$$ l\left(r,t,k\right)=\log p\left({\mathbf{w}}_{t+k}^r|{\mathbf{m}}_r,{\mathbf{w}}_t^r,\dots, {\mathbf{w}}_{t+k-1}^r\right) $$.

Using a sliding window of size *k* to scan all OMIM records, the objective is then to maximize the average log likelihood, as

$$ \frac{1}{T}\sum_{r=1}^m\sum_{t=1}^{l_r-k+1}l\left(r,t,k\right), $$

where *l*_*r*_ is the length of the *r*-th record, and *T* the total number of windows scanned. With a softmax function, the likelihood *p*(*r*, *t*, *k*) is calculated as

$$ p\left({\mathbf{w}}_{t+k}^r|{\mathbf{m}}_r,{\mathbf{w}}_t^r,\dots, {\mathbf{w}}_{t+k-1}^r\right)=\frac{\exp \left({\left({\mathbf{y}}_{t+k}^r\right)}^T{\mathbf{w}}_{t+k}^r\right)}{\sum_{i=1}^w\exp \left({\left({\mathbf{y}}_{t+k}^r\right)}^T{\mathbf{w}}_i\right)}, $$

where the summation is taken over all possible words, and ${\mathbf{y}}_{t+k}^r$ is the predicted vector derived from the record vector **m**_*r*_ and word vectors ${\mathbf{w}}_t^r,\dots, {\mathbf{w}}_{t+k-1}^r$. The *j*-th dimension of ${\mathbf{y}}_{t+k}^r$ is calculated as

$$ {y}_{\left(t+k\right)j}^r={\alpha}_j+{\beta}_j{m}_{rj}+\sum_{i=1}^k{\gamma}_{ij}{w}_{\left(t+i-1\right)j}^r. $$

with *α*, *β* and *γ ‘*s being parameters. Considering that the number of words is typically large (~10^5^), a hierarchical softmax is often adopted for fast training [29]. In order to train the neural network model for the softmax classifier, stochastic gradient descent is often used, and the gradient is obtained by backpropagation. In our study, we default the window size to 5. Our empirical analysis also shows the robustness in the selection of this parameter.

### Derivation of phenotype semantic similarity

Given the vector presentation of disease phenotypes, we characterized semantic similarity between two phenotypes by calculating the cosine of the angle between the corresponding vectors. We also adopted other similarity measures, including Pearson’s correlation coefficient, Spearman’s correlation coefficient, and Euclidean similarity, in the comparative study. Particularly, the Euclidean similarity was transformed from the standard Euclidean distance via a linear transformation to ensure the similarity was in the range of [0,1], while the other three similarity measures were already in such a range according to our numerical analysis. In a similar manner, we characterized the semantic similarity between a phenotype and a gene, and that between two genes.

### Derivation of gene functional similarity

With the RNA-seq data of [33], we characterized a human gene using a 503-dimensional numeric vector that represented logarithm of FPKMs of the gene across the experiments. For a pair of genes indexed by *i* and *j*, we calculated the absolute value of the Pearson’s correlation coefficient of the corresponding vectors to obtain their raw similarity scores ${r}_{ij}^{\left(\mathrm{gexp}\right)}$. We further applied an exponential transformation to convert the raw score into a functional similarity score, as

$$ {s}_{ij}^{\left(\mathrm{gexp}\right)}=\exp \left[-\lambda {\left(\frac{1-{r}_{ij}^{\left(\mathrm{gexp}\right)}}{\sigma_{ij}^{\left(\mathrm{gexp}\right)}}\right)}^2\right], $$

where ${\sigma}_{ij}^{\left(\mathrm{gexp}\right)}$ the standard deviation estimated from raw scores for all pairs of genes, and *λ* a tuning parameter with defaulting value 1.

We calculated pairwise local sequence alignments of human proteins using the Smith-Waterman algorithm implemented in SSEARCH [34]. We then constructed a sequence similarity network of these proteins by connecting two proteins with an undirected edge if their alignment e-value is less than a predefined threshold (10^−4^). Next, we calculated the shortest path distance (${\delta}_{ij}^{\left(\mathrm{gexp}\right)}$) for every pair of proteins *i* and *j* in this network and converted it into a similarity value in the range of 0 and 1 by a linear transformation (${r}_{ij}^{\left(\mathrm{gexp}\right)}=1-{\delta}_{ij}^{\left(\mathrm{gexp}\right)}/\max {\delta}_{ij}^{\left(\mathrm{gexp}\right)}$). Finally, we applied the exponential transformation to convert a raw score to a functional similarity score.

We extracted interactions between proteins from the STRING database (Version 9.1) [35] and constructed a protein-protein interaction network accordingly. Then, as was done for protein sequences, we calculated the shortest path distance (${\delta}_{ij}^{\left(\mathrm{gexp}\right)}$) for every pair of proteins *i* and *j* in this network and converted it into a value in the range of 0 and 1 (${r}_{ij}^{\left(\mathrm{gexp}\right)}=1-{\delta}_{ij}^{\left(\mathrm{gexp}\right)}/\max {\delta}_{ij}^{\left(\mathrm{gexp}\right)}$). Finally, we applied the exponential transformation to convert a row score to a functional similarity score.

We identified 26,784 concepts from the biological process domain of the gene ontology [36] and characterized each human gene using a numeric vector of such number of dimensions. Here, each element in a vector was the information content of the corresponding concept. We calculated the raw similarity score between a pair of genes as the cosine of the angle between the corresponding vectors and applied the exponential transformation to convert a raw score into a functional similarity score.

### Random walk for prioritizing candidate genes

Given a semantic similarity measure for phenotypes, we could construct a nearest neighbor network of diseases by keeping only 10 neighboring diseases of the highest similarity scores for each disease. Similarly, given a functional similarity measure for genes, we could also construct a nearest neighbor network of genes by keeping only 10 neighboring genes of the highest similarity scores for each gene. These two networks, together with known associations between diseases and genes, formed a heterogeneous work that included both diseases and genes and immediately enabled us to adopt the following random walk model for prioritizing candidate genes.

In detail, such a heterogeneous disease-gene network is denoted by a triple **H** = (**D**, **G**, **A**), where **D** = (*d*_*ij*_)_*m* × *m*_ is the weight matrix of the disease subnetwork, **G** = (*g*_*ij*_)_*n* × *n*_ that of the gene subnetwork, **A** = (*a*_*ij*_)_*m* × *n*_ the adjacency matrix of the interconnections, and *m* and *n* the numbers of diseases and genes, respectively. Applying row-normalization to **D**, we obtain a transition matrix **U** = (*u*_*ij*_)_*m* × *m*_, where ${u}_{ij}={d}_{ij}/{\sum}_{j=1}^m{d}_{ij}$ denotes the probability that a random walker moves from the *i*-th disease to the *j*-th disease when it stays in the disease subnetwork. Similarly, we obtain three other transition matrices: **V** = (*v*_*ij*_)_*n* × *n*_ with ${v}_{ij}={g}_{ij}/{\sum}_{j=1}^n{g}_{ij}$ denoting the probability that the walker moves from the *i*-th gene to the *j*-th gene when it stays in the gene subnetwork, **R** = (*r*_*ij*_)_*m* × *n*_ with ${r}_{ij}={a}_{ij}/{\sum}_{j=1}^n{a}_{ij}\kern0.5em \left({r}_{ij}=0\kern0.5em \mathrm{if}\kern0.5em {\sum}_{j=1}^n{a}_{ij}=0\right)$ being the probability that the walker jumps from the *i*-th disease to the *j*-th gene when it stays in the disease subnetwork, and **S** = (*s*_*ij*_)_*n* × *m*_ with ${s}_{ij}={a}_{ji}/{\sum}_{j=1}^m{a}_{ji}\kern0.5em \left({s}_{ij}=0\kern0.5em \mathrm{if}\kern0.5em {\sum}_{j=1}^m{a}_{ij}=0\right)$ being the probability that the walker jumps from the *i*-th gene to the *j*-th disease when it stays in the gene subnetwork. We then define matrix **T** as

$$ \mathbf{T}=\left(\begin{array}{cc}\hfill \left(1-\tau \right)\mathbf{U}\hfill & \hfill \tau \mathbf{R}\hfill \\ {}\hfill \tau \mathbf{S}\hfill & \hfill \left(1-\tau \right)\mathbf{V}\hfill \end{array}\right), $$

and perform row-normalization to obtain the transition matrix for the heterogeneous network as **W** = (*w*_*ij*_)_(*m* + *n*) × (*m* + *n*)_, where ${w}_{ij}={t}_{ij}/{\sum}_{j=1}^{m+n}{t}_{ij}$ and *τ* the probability of jumping from the disease subnetwork to the gene subnetwork or vice versa.

Let ${\mathbf{u}}^{(0)}={\left({u}_i^{(0)}\right)}_{m\times 1}$ and ${\mathbf{v}}^{(0)}={\left({v}_i^{(0)}\right)}_{n\times 1}$ be initial probabilities for the disease and the gene subnetworks, respectively. We obtain **u** ^(0)^ by assigning probabilities proportional to disease similarities to neighbors of the query disease and 0 otherwise, and we set **v** ^(0)^ to zeros to simulate the situation that genetic basis for the query disease is completely unknown. Let **p** ^(0)^ = ((**u** ^(0)^)^*T*^, (**v** ^(0)^)^*T*^)^*T*^ contains initial probabilities for the heterogeneous network and **p** ^(*t*)^ contains probabilities that the walker stays at each node at time *t*, we have the iterative formula

$$ {\mathbf{p}}^{\left(t+1\right)}=\left(1-\pi \right){\mathbf{W}}^T{\mathbf{p}}^{(t)}+\pi {\mathbf{p}}^{(0)}. $$

Solving this linear equation when time tends to infinite, i.e., **p** ^(∞)^ = (1 − *π*)**W** ^*T*^ **p** ^(∞)^ + *π* **p** ^(0)^ with respect to the steady-state probability **p** ^**(∞)**^, we obtain the steady state solution **p** ^(∞)^ = *π*(**I** − (1 − *π*)**W** ^*T*^)^−1^ **p** ^(0)^, which can be decomposed into a disease part ${\mathbf{u}}^{\left(\infty \right)}={\left({u}_i^{\left(\infty \right)}\right)}_{m\times 1}$ and a gene part ${\mathbf{v}}^{\left(\infty \right)}={\left({v}_i^{\left(\infty \right)}\right)}_{n\times 1}$. The later one, **v** ^**(∞)**^, can then be used to score the strength of association between a query disease and candidate genes. It has been show that the random walk model is not sensitive to the parameters involved in the model [17]. We therefore set default values for the parameters as τ = 0.5, π = 0.7 and ε = 10^−4^.

## Abbreviations

ACC: Classification accuracy
AUC: Area under the receiver operating characteristic curve
BER: Balanced error rate
BOW: Bag-of-words
DAG: Directed acyclic graph
HPO: Human phenotype ontology
IDF: Iinverse document frequency
LR: Logistic regression
MeSH: Medical subject headings
MIM: Mendelian inheritance in man
MRR: Mean rank ratio
OMIM: Online Mendelian inheritance in man
PC: Principle component
PCA: Principle component analysis
RF: Random forest
SVM: Support vector machine
TF: Term frequency
TF-IDF: Term frequency-inverse document frequency
TOP: Top ranked test genes
UMLS: Unified medical language system
